# Editorial: Adaptive immunity to respiratory pathogens

**DOI:** 10.3389/fimmu.2023.1174178

**Published:** 2023-03-06

**Authors:** Naoki Iwanaga, Priyadharshini Devarajan, Anukul T. Shenoy

**Affiliations:** ^1^ Department of Respiratory Medicine, Nagasaki University Hospital, Nagasaki, Japan; ^2^ Department of Pathology, University of Massachusetts Chan Medical School, Worcester, MA, United States; ^3^ Pulmonary Center, Boston University Chobanian & Avedisian School of Medicine, Boston, MA, United States

**Keywords:** respiratory infection, adaptive immunity, lung immunity, vaccines, tissue resident immune cells, lung microenvironment, aging, SARS – CoV – 2

Adult humans inhale ~11,000 liters of air harboring irritants and harmful airborne pathogens daily. Thus, the lungs forming a direct interface between the circulation and external environment play a crucial role in eliminating or containing these pathogens while maintaining efficient gas exchange and host survival. This is accomplished through collaboration between innate and adaptive immune systems in the lung. Tissue resident subsets of the adaptive arm include CD4^+^ and CD8^+^ T_RM_ cells (1), and B_RM_ cells (2, 3) that are integral to immunity against respiratory pathogens.

The SARS-CoV-2 pandemic has accelerated respiratory immunology and vaccine research. Vaccines have continued to remain effective against severe disease caused by continuously emerging variants, pointing to the crucial roles played by T cells (4). Sieber at al. here strengthen the evidence for this, by showing that while SARS-CoV2 infection with the ancestral strain (D614G isolate) in children induced a robust and lasting neutralizing antibody response (for upto 12 months post infection) to the infecting strain, the ability of these D614G-neutralizing antibodies to neutralize the omicron isolate was significantly reduced. Instead it was predominantly the CD4^+^ followed by the CD8^+^ T cells induced by the ancestral D614 isolate that maintain broad reactivity to the omicron variant. Natural infection-induced protection seemed to be longlasting and broadly crossprotective primarily *via* T lymphocytes. It is tempting to speculate that the severity of inflammatory milieu created in a natural infection plays a critical role in inducing such robust memory recall infections. Consistent with this, and extending this concept to B cells, Graninger et al. demonstrate that adult individuals that were hospitalized for severe natural infection with SARS-Co-V2 had more robust and broadly cross-neutralizing antibodies to the ancestral D614G isolate, the beta and delta variants, when compared to patients that were not hospitalized for their SARS-Co-V2 infection. It is likely that severe natural infection can remodel host immune landscape to bolster cross-reactive protection. It is well known that while vaccines elicit immune responses against primarily the epitopes in the vaccine (spike protein for most COVID-19 vaccines), natural infection elicits responses against many viral epitopes; thus increasing the breadth of the response. T cells target internal viral epitopes that are less prone to mutation and are thus key to widely discussed cross-protection against mutating strains (4). These, T cell responses are more strongly elicited by natural infection because of the long-lived antigen presentation induced by live infection alongwith the host of supporting innate responses (5, 6). Lung adaptive immunity also cannot exert its protective effects without help from local innate immune and stromal cells. T cell immunity needs antigen presentation by monocytes (7) and epithelial cells (8) for establishing optimal lung residency while requiring help from epithelial (9) and fibroblasts (10) to recruit antimicrobial effectors. Further, stromal cells express and secrete factors to maintain the tertiary lymphoid architectures within the lungs (11, 12). Nevertheless, both studies reported herein consistently show that vaccination of previously uninfected individuals induced antibodies with higher neutralizing capacity to the ancestral isolate and the newer variants compared to unvaccinated individuals. Moreover, protection from vaccines comes without the lung tissue pathology caused by live infection that can sometimes lead to hospitalization and death. Keuning et al. here show that saliva-based antibody assays can measure SARS-CoV-2 humoral immunity with high confidence without the need for invasive blood sampling; a finding that can simplify longitudinal analysis of antibody levels in human cohorts and allow identification of vulnerable populations.

While the pandemic has provided a rare window into rapid advancement of directly translational immunological findings, it revealed several gaps in our knowledge about immunity to respiratory infections and the design of ideal vaccines.

## Inducing effective vaccine derived protection

The new generation of mRNA vaccines induce strong protection against rapidly mutating virus compared to conventional vaccines. Graninger et al. find that healthy individuals vaccinated with two doses of BNT162b2 consistently showed better neutralizing antibody titers when compared to the patients that were naturally infected with SARS-Co-V2 but did not need hospitalization. A mechanism could be that liposomes and the RNA molecules strongly adjuvant mRNA vaccines and present antigen for prolonged periods of time compared to conventional protein based vaccines (13, 14). mRNA vaccines also induce strong T cell responses [which may include T_FH_ cells known to provide better help to B cells (6)] and hence have long been recognized as essential for “universal vaccines” in the influenza field (5). Intranasal vaccines that induce local immunity in the respiratory tract, have now garnered more attention as key to bolstering frontline immunity early against respiratory infections including SARS-CoV-2 (15, 16). In this issue, Hassert and Harty, and Hirai and Yoshioka comprehensively review our current state of knowledge on CD4^+^ and CD8^+^ T cells protection against respiratory pathogens and provide perspectives on exploiting their cross-reactivity in rational vaccine design.

## Trade-off between protection vs pathology

While able to provide robust protection, lung T cell responses can perturb the delicate balance between protective immunity vs tissue damage in a sensitive vital organ like the lung. This, again leads us to the question – are highly potent lung localized responses more of a detriment to protection/recovery from infection? Hirai and Yoshioka here comprehensively review our knowledge regarding this trade-off.

## Disparities in vaccine efficacy and infection induced hospitalizations/death in the aged

Lower respiratory tract infections (LRTI) lead to the majority of hospitalizations in people over the age of 65 (17). Naive T and B cell responses in the aged wane and they predominantly depend on previously established memory to respond to new infections and vaccines. Thus, it is essential to understand how memory established years ago can contribute to protection, while also studying other causes that lead to poor immunity in the aged. In this issue, Torrance and Haynes review how aging and senescence in the innate and adaptive immune compartments of the lungs (and their dysregulated intercellular crosstalk) increases acute and chronic susceptibility of the aged to respiratory diseases, and discuss the use of senolytics in improving the aged immune response.

## Boosting waning immune responses vs inducing original antigenic sin

As real-world questions about booster vaccines arise, it also rekindles questions about “original antigenic sin” – the loss of our immune system’s ability to respond to new variants because of existing immunity to immunodominant epitopes (18). Work by Sieber at al. published in this issue, may however contradict this notion of OAS and strengthen current evidence in the COVID-19 field by showing that immunization of ancestral SARS-CoV2 recovered children with BNT162b2 or Ad26.COV2-S vaccines boosted broadly neutralizing abilities of serum antibodies beyond the original antigenic strain which in this case was the omicron variant. Nevertheless, more studies testing the veracity and the conditions in which OAS become relevant and understanding of the effects of repeated vaccination not only on B- and T- cell responses, but also on the lymphocyte niche warrants more investigation.

## Author contributions

All authors listed have made a substantial, direct, and intellectual contribution to the work and approved it for publication.
